# Comparing the Level of Pain and Oral Hygiene Status in Patients Receiving Fixed Appliance Therapy Versus Clear Aligner Therapy During the First Phase of Orthodontic Treatment: An In Vivo Study

**DOI:** 10.7759/cureus.91700

**Published:** 2025-09-06

**Authors:** Rhoshan B, Suresh Babu C, Moosa Ahmed P, Jithesh Kumar K, Panjami Mearish, Steve Mathew Jacob, Aravind Haridas

**Affiliations:** 1 Department of Orthodontics and Dentofacial Orthopedics, Mahe Institute of Dental Sciences and Hospital, Pondicherry University, Mahe, IND; 2 Department of Orthodontics, Mahe Institute of Dental Sciences and Hospital, Pondicherry University, Mahe, IND; 3 Department of Orthodontics and Dentofacial Orthopedics, Mahe institute of Dental Sciences and Hospital, Pondicherry University, Mahe, IND

**Keywords:** aligners, clear aligner, fixed appliance therapy, hygiene, malocclusion, orthodontics, therapy

## Abstract

Background: Patients' compliance with orthodontic treatment, particularly in the first phases of therapy, is influenced by the perception of pain and oral hygiene management. Even though clear aligners and fixed orthodontic appliances have the same popularity, their comparative impacts on these early treatment parameters are not well researched.

Aims: The study's intention was to contrast the level of oral hygiene and discomfort experienced by patients undergoing clear aligner therapy at an early stage of orthodontic treatment with those undergoing fixed appliance therapy.

Methodology: Forty patients were gathered and split into two groups at random. Group I was required to wear fixed orthodontic appliances, whereas group II received clear aligners. Age, gender, and the degree of malocclusion were all matched between the groups. The degree of pain was measured using the visual analog scale (VAS) and the Wong-Baker FACES Pain Rating Scale. To evaluate oral hygiene, the Simplified Oral Hygiene Index (OHI-S) was used. Baseline homogeneity was guaranteed by the Index of Complexity, Outcome, and Need (ICON) and Little's Irregularity Index. T-tests and Mann-Whitney U tests (p < 0.05) were used in the statistical analysis.

Results: The scores on pain were low (3.89, 2.16), and oral hygiene was improved (mean OHI-S = 1.92, 1.06) in those who had clear aligner treatment, but these did not show a significant difference when compared to those who had fixed orthodontic appliances.

Conclusion: Clear aligners potentially provide better patient comfort and allow better oral hygiene in early orthodontic treatment, which makes them a preferable choice for initial patient compliance.

## Introduction

The goal of orthodontic treatment is to treat malocclusions and skeletal craniofacial abnormalities and optimize their function and appearance [1]. Fixed or removable orthodontic appliances may have a severe influence on periodontal tissues and oral microbiota, and it is necessary to evaluate the biological effects of these appliances [2]. Pain is a very subjective phenomenon and may differ significantly across individuals, even in the face of similar stimuli [3]. Various attributes affect the perception of pain during treatment, including age, gender, emotional status, pain threshold, strength of the force applied, and previous experiences [4]. Orthodontic procedures are the most common complaint of pain, and pain directly affects patient satisfaction [5]. It can even result in termination of treatment in certain cases [6]. It is commonly understood that using fixed appliances leads to a high level of pain after 24 hours of archwire insertion, which gradually declines during the following days [7]. Even the kind of appliance applied may affect not only the level of discomfort but also the patient's capacity to preserve oral hygiene [8]. The structure of dental plaque is changed using fixed appliances, whereas removable aligners enable the intermittent application of force, which can help recover tissues and minimize the presence of biofilm [9].

Fixed orthodontic appliances made of stainless steel or ceramic materials are common and cost-effective, and have had a long history of clinical success [10]. Nevertheless, they are commonly linked to discomfort, especially in the initial stages of therapy. Clear aligners have become a new alternative, which are more comfortable, aesthetically better, and easier to use, especially with patients who have high aesthetic expectations and are adults [11]. Nevertheless, despite these benefits, pain is one of the determinants of treatment satisfaction [12]. Reports indicate that, when contrasted with fixed orthodontic appliances, clear aligners lessen the sense of suffering, particularly in the first stages of therapy [13]. However, other studies have recorded neutral, and in some cases, conflicting results [14]. Invisalign (Align Technology, San Jose, CA) patients have demonstrated similar overall standard of living outcomes as patients with fixed braces, but superior outcomes were seen in other areas, including eating and chewing among aligner wearers [15]. Because of the lack of and inconsistency in evidence, this study's objective is to assess oral hygiene and discomfort levels of individuals receiving fixed appliance therapy with those receiving clear aligner therapy at the start of orthodontic therapy.

Objectives of the study

The key focus of the investigation is to contrast the degree of dental hygiene and pain perception between individuals receiving therapy with permanent appliances and those undergoing Lucid Aligner therapy at the most advanced stage of orthodontic treatment. The intended in vivo study will help figure out whether clear aligners provide a quantifiable benefit over conventional fixed appliances in regard to patient-reported discomfort and preserving oral health.

## Materials and methods

Study design and ethical approval

The present clinically controlled randomized investigation was undertaken from August 2024 to December 2024 at the Department of Orthodontics of Mahe Institute of Dental Sciences & Hospital. The Institutional Ethics Committee of Mahe Institute of Dental Sciences & Hospital has granted ethical clearance (Ref. No. MINDS/PG-ETHICAL/013/2023-24) for this study. All participants provided informed consent before enrollment.

Quantity of samples

G*Power version 3.1 (Heinrich Heine University Düsseldorf, Düsseldorf, Germany) was utilized to compute the sample size. A prior study that contrasted fixed orthodontic appliances and aligner-based therapy in terms of pain perception and dental hygiene yielded the effect size (Cohen's d = 0.65) [3]. Each group required a minimum of 17 samples at 0.05 alpha and 80% power. To accommodate a 10% attrition rate, 20 participants per group were included.

Sample size determination was established through power analysis, ensuring 80% power at a 95% confidence level. With the consideration of the estimated attrition rate of 10%, the corrected final sample size became 17 participants in each group (15 + 10% of 17).

Study sample

The sample size used in the study was 40 people who were split into two equal groups to be treated. The study sample was divided into two groups: 20 patients who had received fixed appliance therapy (FAT) and 20 patients who had undergone clear aligner therapy (CAT).

Eligibility criteria

Potential confounding factors considered in this study included age, gender, baseline oral hygiene status, brushing methods, dietary habits, smoking, systemic conditions, pain threshold variability, and prior experience receiving orthodontic treatment. These were controlled through age and gender matching across groups, inclusion of only those subjects with good baseline oral hygiene (Simplified Oral Hygiene Index ≤ 1), exclusion of individuals with deleterious habits or systemic disease, and provision of standardized oral hygiene instructions by the same operator. In addition, the same brand of brackets and aligners was used to minimize appliance variability, and participants were randomly allocated into groups to reduce selection bias. Participants enrolled while undergoing non-extraction orthodontic care for the maxillary and mandibular dental arches were above the age of 18, and were in good oral and general health. Participants with systemic medical illnesses, missing teeth that needed prosthetic rehabilitation, untreated dental caries, or periodontal disease were not considered for inclusion.

Group matching

During the recruitment period, to ensure comparability between the fixed orthodontic appliance group and the clear aligner therapy group, participants in the latter were matched within a range of ±3 years to those in the former. Before enrollment, all participants were briefed on the research aims and objectives and protocols, and informed consent documentation was signed and obtained. The protocol adhered to the ethical standards set forth by the institution.

Outcome measures

Pain was assessed using two validated self-reported instruments: the visual analog scale (VAS) [4] and the Wong-Baker FACES Pain Rating Scale [9]. Both scales were administered at baseline (before appliance placement) and repeated at 24 hours, 72 hours, and two weeks following appliance placement to capture changes in pain over time. Oral hygiene status was evaluated clinically using the Simplified Oral Hygiene Index (OHI-S) [5] at baseline and again at two weeks, coinciding with the short-term follow-up period. To determine the initial severity of malocclusion, the Index of Complexity, Outcome, and Need (ICON) [12] and Little’s Irregularity Index [10] were recorded once at baseline before treatment initiation. Thus, pain was measured longitudinally at multiple time points, oral hygiene was assessed at two intervals (baseline and two weeks), and malocclusion severity was evaluated once at baseline.

Baseline group comparability

Age, gender distribution, and baseline malocclusion severity were all matched between the two groups. This ensured a baseline for assessing the disparities in pain perception and oral outcomes of the treatment modalities. The scoring system applied to assess how severe malocclusion is presented in Table 1 and depends on clinical data, i.e., crowding, spacing, overbite, crossbite, and relationships of the buccal segments. The scores are between 0 and 5 and assist in determining treatment needs and complexity.

**Table 1:** Orthodontic treatment need and malocclusion severity scoring criteria. The described scores were analyzed using ANOVA. IOTN-AC: Index of Orthodontic Treatment Need - Aesthetic Component.

Score	0	1	2	3	4	5
Aesthetic	1–10, as judged using IOTN-AC	—	—	—	—	—
Upper arch crowding	Less than 2 mm	2.1–5 mm	5.1–9 mm	9.1–13 mm	13.1–17 mm	>17 mm or impacted teeth
Upper arch spacing	Up to 2 mm	2.1–5 mm	5.1–9 mm	>9 mm	—	—
Crossbite	No crossbite	Crossbite present	—	—	—	—
Incisor overbite	Up to 1/3 tooth coverage	1/3–2/3 coverage	2/3 up to full coverage	Full coverage	—	—
Sagittal relationship of the buccal segment	Left and right added together	Cusp to embrasure relationship only (Class I, II, or III)	Any cusp relationship up to but not including cusp to cusp	Cusp to cusp relationship	—	—

Quantitative analysis

The statistical evaluation was conducted using IBM SPSS Statistics software, version 26.0 (IBM Corp., Armonk, NY). The demographic and clinical features were presented in tables and graphs and described using descriptive statistics. An independent samples t-test was applied to compare the OHI-S scores between the two groups. Since pain perception scores did not follow a normal distribution, group differences were evaluated using the Mann-Whitney U test. The threshold for statistical significance was defined as p ≤ 0.05. Normality of the data distribution was assessed using the Shapiro-Wilk test prior to selecting the appropriate statistical test (parametric or non-parametric).

## Results

Status of oral hygiene (OHI-S comparison)

The average OHI-S was improved in the fixed appliance therapy group (2.58 + 1.14) than in the clear aligner therapy group (1.92 + 1.06), indicating better oral hygiene within the aligner cluster. Yet, this variation was not statistically significant (t = 1.853, p = 0.072). As per the OHI-S classification, all subjects of both groups showed fair oral hygiene (score range: 1.303.0). Table 2 presents the results of comparing the groups' oral hygiene status.

**Table 2:** Comparison of treatment groups (fixed appliance vs. clear aligner). OHI-S: Simplified Oral Hygiene Index.

Variable	Group I: Fixed appliance (mean ± SD)	Group II: Clear aligner (mean ± SD)	t/U value	p-value
OHI-S	2.58 ± 1.14	1.92 ± 1.06	t = 1.853	0.072
Pain Index (Wong-Baker)	4.21 ± 1.75	3.89 ± 2.16	t = 0.495	0.623
Little’s Index	2.47 ± 0.96	2.47 ± 0.96	t = 0.000	1.000

**Figure 1:**
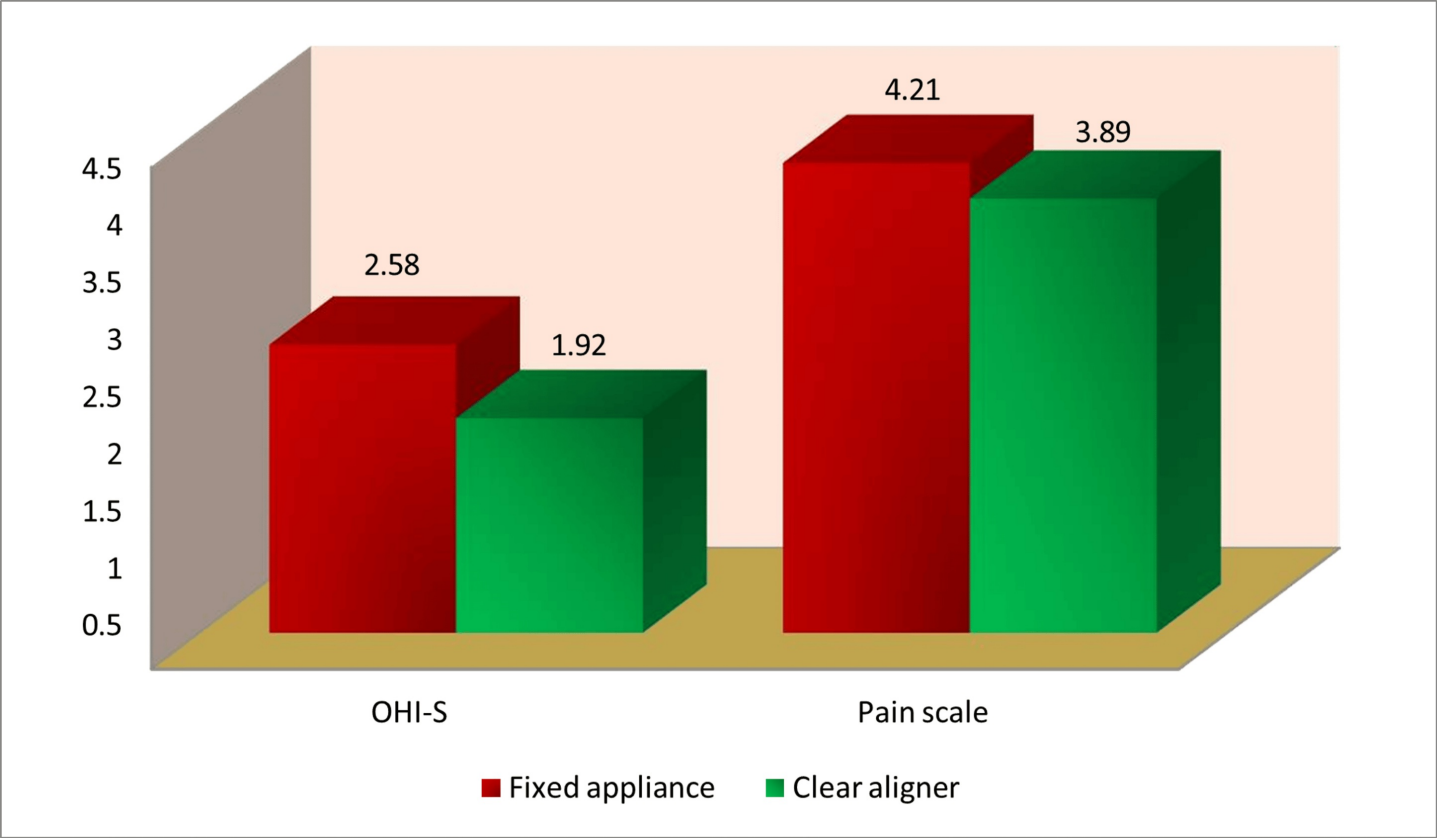
Comparison of mean OHI-S and pain scores between treatment groups. OHI-S: Simplified Oral Hygiene Index.

Figure 1 shows the average pain perception and oral hygiene (OHI-S) ratings for the patients assigned to fixed appliance or clear aligner therapy cohorts. The mean values for both parameters decreased as a result of clear aligner therapy.

Pain perception

**Figure 2:**
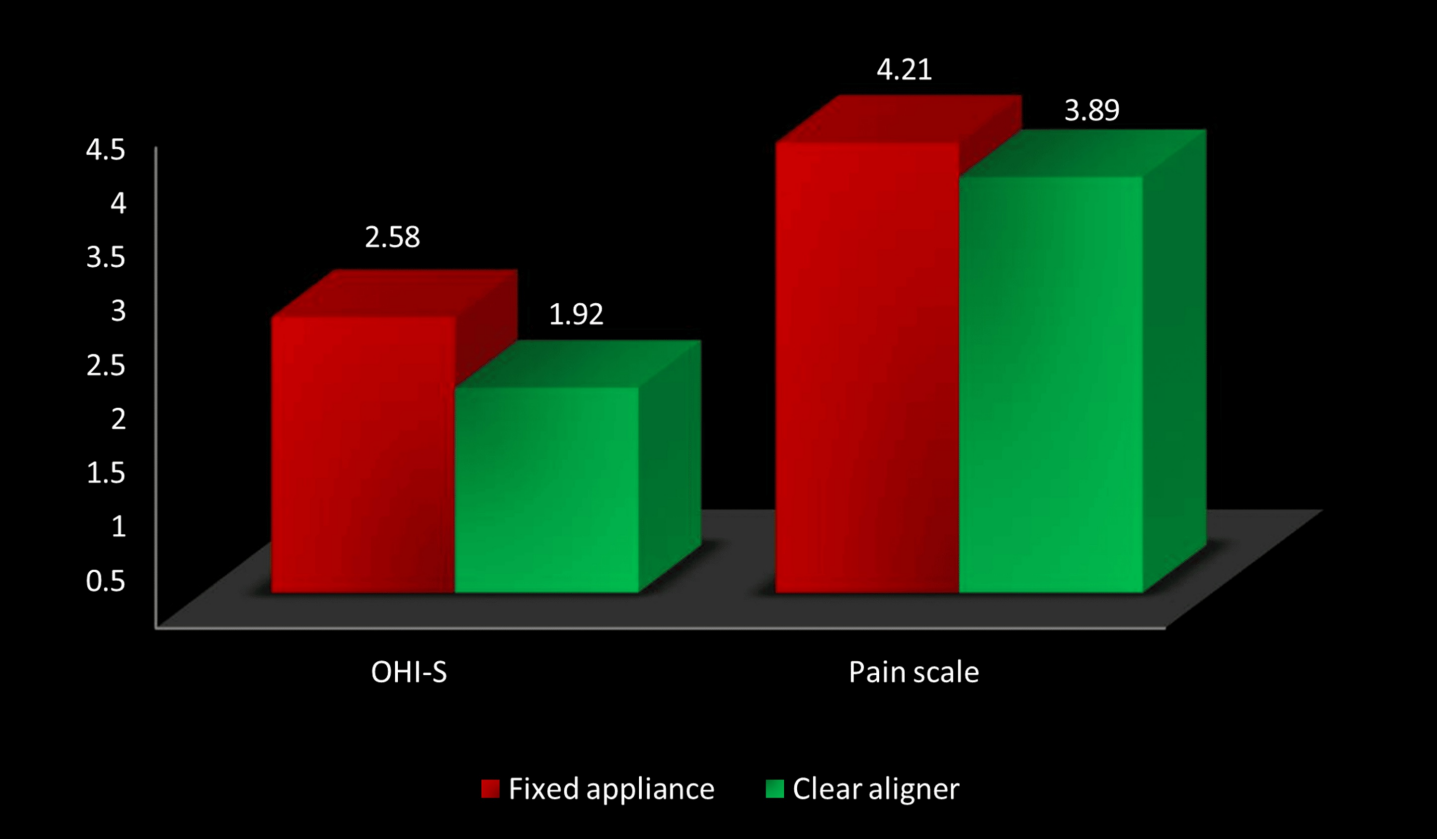
Mean OHI-S and pain scores for fixed appliance vs. clear aligner therapy. OHI-S: Simplified Oral Hygiene Index.

The Wong-Baker FACES Pain Rating Scale showed a slight difference in pain perception (3.89, 2.16) in the group receiving fixed appliances and the clear aligner therapy group (4.21, 1.75). There was no statistical significance in this (t = 0.495, p = 0.623). Figure 2 illustrates the comparison of oral hygiene status (OHI-S) and pain perception in of treatment groups. Clear aligner therapy had a lower mean score on both parameters, which means a higher level of oral hygiene and fewer discomforts; however, there was no statistically significant change.

In addition to the quantitative findings, representative intraoral photographs were included to visually document the early treatment progress in both groups.

Intraoral documentation of treatment progress

**Figure 3:**
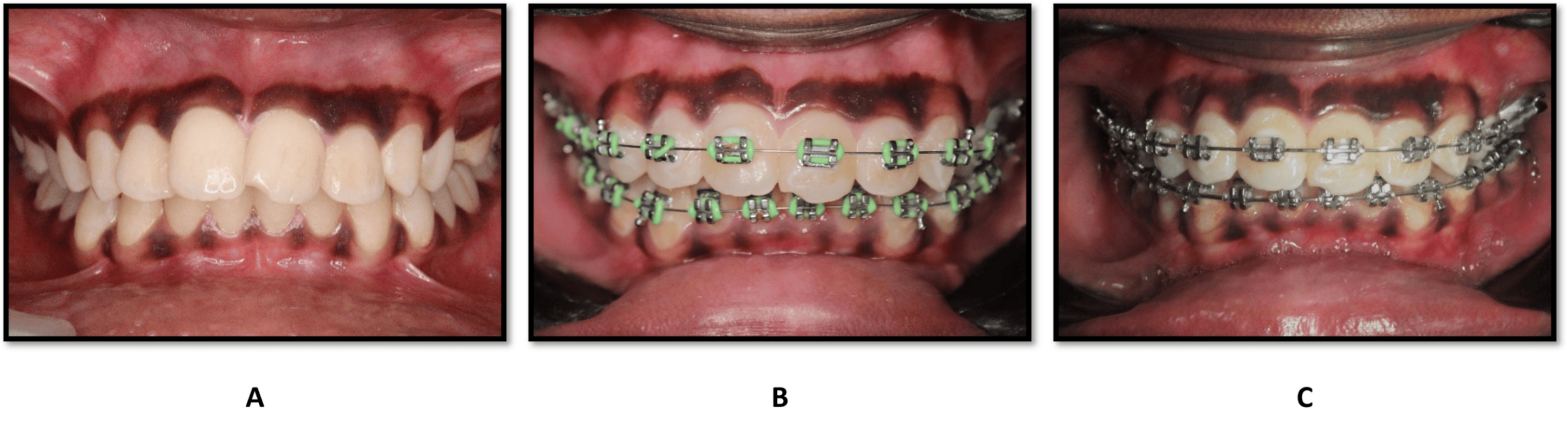
Fixed appliance therapy. (A) Pre-treatment intraoral frontal view. (B) On bonding with the initial archwire. (C) After two weeks of treatment.

**Figure 4:**
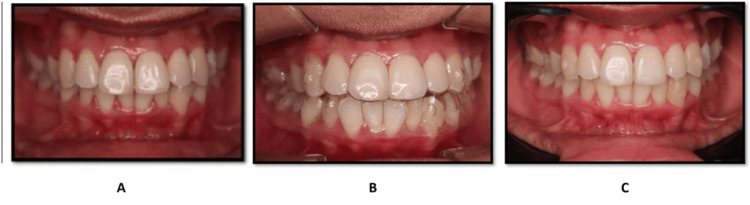
Clear aligner therapy. (A) Pre-treatment intraoral frontal view. (B) With the first clear aligner tray. (C) After two weeks of treatment.

Figures 3, 4 represent intraoral images of patients receiving fixed appliance therapy and clear aligner therapy, respectively. For each group, pictures are presented at the baseline (pre-treatment), when the appliances are placed, and at the end of two weeks of treatment. These images illustrate qualitatively the early intraoral presentation and treatment progression with the observed quantitative results.

Level of malocclusion

Baseline malocclusion severity was similar in the two groups, with the mean Little's Irregularity Index being the same (2.47 0.96). Table 1 shows that the differences between the groups were not found to be statistically significant (t = 0.000, p = 1.000).

Index of Complexity, Outcome, and Need (ICON) classification

To further determine the difficulty of the initial malocclusion, the patients were categorized using the ICON. The two treatment groups had identical distributions: 15.8% for easy, 36.8% for mild, 31.6% for moderate, and 15.8% for difficult. The distribution of ICON categories among the groups did not show any significant variation in statistical terms, according to the chi-square test (t = 0.000, p = 1.000). The ICON classification of malocclusion severity is displayed in Table 3, which demonstrates equal distribution throughout the treatment groups and no discernible variation.

**Table 3:** ICON classification of malocclusion severity across treatment groups. ICON: Index of Complexity, Outcome, and Need.

Group	Frequency type	Easy (1)	Mild (2)	Moderate (3)	Difficult (4)	Total
Fixed appliance	Count	3	7	6	3	19
Fixed appliance	% within group	15.8%	36.8%	31.6%	15.8%	100%
Clear aligner	Count	3	7	6	3	19
Clear aligner	% within group	15.8%	36.8%	31.6%	15.8%	100%
Total	Count	6	14	12	6	38
Total	% within group	15.8%	36.8%	31.6%	15.8%	100%
Statistical test	Pearson chi-square	χ²(3, N = 38) = 0.000, *p* = 1.000	χ²(3, N = 38) = 0.000, *p* = 1.000	χ²(3, N = 38) = 0.000, *p* = 1.000	χ²(3, N = 38) = 0.000, *p* = 1.000	χ²(3, N = 38) = 0.000, *p* = 1.000

## Discussion

The recent increased popularity of clear aligner therapy in the past few years is suggestive of a more significant shift in patient desire for more aesthetic and pleasing orthodontic treatments [16]. Unlike fixed braces, clear aligners can be removed, are transparent, and are usually associated with minimal disturbance of the lifestyle of the patient, especially adults who demand a discreet treatment [17]. The current study's results are consistent with the trend, as the group using clear aligners reported better dental hygiene and less pain than the group wearing permanent appliances [18]. The increased patient satisfaction with clear aligners, in particular, chewing, speech, and social confidence, has also been mentioned in some of the previous studies [19]. This is justified by the fact that the aligners are smoother and customized, and hence they cause less irritation to the mucosa and disturbance of the oral functions [20]. Nevertheless, although clear aligners have many benefits, they are less efficient at managing intricate movements than permanent appliances, including root torque, rotation, and vertical intrusion [21].

Fixed appliances still provide better biomechanical control and are therefore the appliances of choice in moderate to severe malocclusions [22]. With that said, the brackets, bands, and archwires utilized in fixed systems have been linked to a higher risk of plaque accumulation [23]. These appliances provide more surfaces to harbor biofilm and make daily oral hygiene practices more difficult [24]. The association between inability to implement oral hygiene and fixed appliances is not a secret, and the literature shows an increased prevalence of gingival inflammation and enamel demineralization during the period of treatment [25]. Conversely, the removability of clear aligners enables patients to continue their regular brushing and flossing, which minimizes periodontal complications [26]. Moreover, the aligners are removable at mealtimes, which helps to evade numerous dietary limitations that accompany fixed appliances [27].

Suffering is one of the most frequently mentioned issues during orthodontic therapy, and it can be experienced in 95% of patients [28]. It normally reaches its peak in less than 24 hours following the appliances' activation, and it decreases over the days as the tissues become accustomed to the forces exerted [13]. The primary causes of this pain are the release of inflammatory mediators and mechanical strain on the periodontal ligament [29]. Orthodontic therapy may also have psychological and emotional effects on how pain is perceived [8]. The subjects of clear aligner treatment in the present research experienced fewer pains, which corroborates the research that states fewer pains during the initial stages of treatment with aligners [30]. This may be attributed to the gentler and more consistent forces applied by clear aligners, as well as the absence of protrusive components such as brackets and ligatures, which often contribute to mucosal irritation and discomfort [31].

It should be noted, though, that not every study supports this finding. There has been some evidence showing that patients who use clear aligners or lingual appliances can feel even more or as much pain in the early phases of the treatment [32]. This can be attributed to personal pain thresholds, complexity of the case, or variations in treatment procedures [28]. Dietary limitations are another important consideration in orthodontic care. The use of appliances that are fixed could result in eating problems, especially in the early adaptation phase [33]. Patients often complain about a change in the chewing pattern and a decrease in satisfaction with the food intake [29]. On the contrary, the eating-related issues with clear aligners are usually minor and reflect positively on their overall satisfaction with treatment [12]. In the present investigation, the baseline malocclusion severity of the two treatment groups was similar using both the ICON and Little indices, ruling out the initial complexity of cases as the reason behind the differences in outcomes [34]. This supports the validity of the differences in the perception of pain and oral hygiene that were observed in the two groups.

Clinical implications

According to the study, clear aligners offer advantages with respect to reduced pain perception and improved oral hygiene in the early phases of orthodontic therapy, thereby enhancing comfort and compliance of patients. Since the differences were not statistically significant, appliance selection should still be based on malocclusion severity, treatment goals, and patient preference rather than comfort and hygiene outcomes alone.

Limitations

The short-term evaluation and small sample size of this study were its limitations, which reduce the generalizability of results. In addition, pain assessment relied on subjective self-reported measures, potentially subject to bias. The rigorous estimation of the sample size using the previous effect size, along with strict control of confounders such as age, gender, baseline oral hygiene, and pain threshold variability, strengthens the internal validity of the present study and ensures that observed differences can be ascribed to the therapy modality rather than external factors.

## Conclusions

The proposed study offers a specific comparison of the level of pain perception and the degree of oral hygiene of fixed appliances and clear aligners in the context of the first phase of orthodontic treatment, which is not much studied, but significantly contributes to the formation of adherence and satisfaction of the patient. As opposed to more general assessments of treatment outcomes, the study isolates the initial patient response, when discomfort and hygiene issues are normally most pronounced, and provides controlled data through matched cohorts with similar levels of malocclusion. Such a limited timeline and controlled study design make the results especially applicable to clinicians who want to optimize the early patient experience and reduce the chance of early dropout or compliance problems.

Filling in the literature gap in orthodontics where previous research frequently provides contradictory findings or does not allow the detailed comparison of the results confined to the initial stage of adjustment, the study adds clarity to the direct impact of the appliance choice on patient-reported outcomes. This evidence indicates that clear aligners might have some practical short-term benefits, including oral hygiene comfort and ease, which are vital to patient morale and involvement in the initial stages. The practical implications of these insights to clinical decision-making are that, although it is important to select appliances based on biomechanical requirements, it is also important to consider patient-centered factors at the very beginning of treatment to tailor the appliance selection to the patient.
